# Neutrophil Elastase Inhibitors Suppress Oxidative Stress in Lung during Liver Transplantation

**DOI:** 10.1155/2019/7323986

**Published:** 2019-11-23

**Authors:** Weifeng Yao, Xue Han, Yu Guan, Jianqiang Guan, Shan Wu, Chaojin Chen, Haobo Li, Ziqing Hei

**Affiliations:** ^1^Department of Anesthesiology, The Third Affiliated Hospital, Sun Yat-sen University, Guangzhou, Guangdong 510630, China; ^2^Department of Anesthesiology, Sun Yat-sen Memorial Hospital, Sun Yat-sen University, Guangzhou 510000, China; ^3^Department of Anesthesiology, Affiliated Hospital of Guangdong Medical University, Zhanjiang, Guangdong, China

## Abstract

**Background:**

Neutrophil infiltration plays a critical role in the pathogenesis of acute lung injury following liver transplantation (LT). Neutrophil elastase is released from neutrophils during pulmonary polymorphonuclear neutrophil activation and sequestration. The aim of the study was to investigate whether the inhibition of neutrophil elastase could lead to the restoration of pulmonary function following LT.

**Methods:**

In *in vivo* experiments, lung tissue and bronchoalveolar lavage fluid (BALF) were collected at 2, 4, 8, and 24 h after rats were subjected to orthotopic autologous LT (OALT), and neutrophil infiltration was detected. Next, neutrophil elastase inhibitors, sivelestat sodium hydrate (exogenous) and serpin family B member 1 (SERPINB1) (endogenous), were administered to rats before OALT, and neutrophil infiltration, pulmonary oxidative stress, and barrier function were measured at 8 h after OALT.

**Results:**

Obvious neutrophil infiltration occurred from 2 h and peaked at 8 h in the lungs of rats after they were subjected to OALT, as evidenced by an increase in naphthol-positive cells, BALF neutrophil elastase activity, and lung myeloperoxidase activity. Treatment with neutrophil elastase inhibitors, either sivelestat sodium hydrate or SERPINB1, effectively reduced lung naphthol-positive cells and BALF inflammatory cell content, increased expression of lung HO-1 and tight junction proteins ZO-1 and occludin, and increased the activity of superoxide dismutase.

**Conclusion:**

Neutrophil elastase inhibitors, sivelestat sodium hydrate and SERPINB1, both reduced lung neutrophil infiltration and pulmonary oxidative stress and finally restored pulmonary barrier function.

## 1. Introduction

To date, liver transplantation (LT) is the most effective clinical therapy for end-stage liver disease [1, 2]. Although the one-year survival rate for LT recipients is 90%, early complications, especially acute lung injury (ALI), are still life-threatening, with mortality exceeding 50% after progression to acute respiratory distress syndrome [3, 4]. Extended cold storage and graft injury induced by ischemia/reperfusion are documented to contribute to the occurrence of ALI [5–7]. However, the cross-talk mechanism of the liver-lung axis remains unknown.

Neutrophils are circulating peripheral blood leukocytes, which can be rapidly recruited not only to an infection site against various pathogens, but also to sites of injury and inflammation [8, 9]. It is generally thought that in the case of aseptic inflammation, neutrophils are recruited to the site of inflammation and cause further damage before eventually being swallowed and degraded by macrophages [10, 11]. However, Wang et al. recently found that neutrophils enter the site of liver damage after thermal cauterization using a liver thermal burn model, which leads to the removal of injured blood vessels and rebuilding of new blood vessel channels. Interestingly, this group of neutrophils that enters the injury site neither dies nor is swallowed by macrophages but instead returns to the blood vessels and enters the lungs and bone marrow, indicating that neutrophils may play a critical role in the liver-lung axis when the liver is suffering from damage [12]. However, the damage mechanism of neutrophil reverse migration from liver to lung during LT remains unknown.

Neutrophil elastase (NE), mainly released from neutrophils, plays a critical role in endothelial injury during ALI [13, 14]. Uchida et al. showed that NE might cause rat liver damage early after reperfusion, indicating that neutrophils may be activated and may start to release NE in the liver during LT [15]. Moreover, in our previous study, we found that NE level was significantly elevated in bronchoalveolar lavage fluid (BALF) after rats were subjected to LT, indicating that NE may be an important mediator in the liver-to-lung axis during LT [16]. And we found that overexpression of the endogenous NE inhibitor SERPINB1 could restore pulmonary function from LT. However, there is no biological agent for SERPINB1 at present. Sivelestat sodium hydrate is another NE inhibitor, which is a new small molecule NE inhibitor. Whether sivelestat sodium hydrate can play a role in lung protection similar to SERPINB1 is unknown.

Here, in the current study, we designed an animal study to explore the role of sivelestat sodium hydrate in lung protection compared to SERPINB1. We hypothesized that the inhibition of NE by novel synthetic NE inhibitors, sivelestat sodium hydrate, could lead to the attenuation of ALI and the restoration of pulmonary function after LT.

## 2. Methods and Materials

### 2.1. Animals

Healthy SPF (Sprague-Dawley, SD) male rats (200–250 g) were purchased from the Experimental Animal Center of Guangdong Province. Rats were exposed to room temperature of 25-27°C and provided basic feed for one week before conducting experiments. All the animal care and experimental protocols in the current study were approved by the Institutional Animal Care and Use Committee of Sun Yat-sen University (Guangzhou, China) and performed in accordance with National Institutes of Health guidelines (National Institutes of Health publication 86-23 revised 1985) for the use of experimental animals.

### 2.2. Experiment Design

Firstly, rats were randomly subjected to sham operation or orthotopic autologous liver transplantation (OALT) surgery. Samples were collected at different timepoints after liver reperfusion: 4 h (T4 group, *n* = 6), 8 h (T8 group, *n* = 6), 16 h (T16 group, *n* = 6), 24 h (T24 group, *n* = 6), and 48 h (T48 group, *n* = 6). OALT surgery was established in our lab and performed as previously described [16–19]. The total ischemic time of the liver transplant is about 30 minutes. Equilibrium liquid was given through infusion according to the volume of blood loss (about 1 mL) during the surgery. Rats receiving sham operation underwent laparotomy, and their blood vessels were separated after anesthesia. Rats receiving sham operation did not have liver ischemia and did not undergo reperfusion.

Next, rats were randomly divided into four groups (*n* = 6 per group) to observe the effects of neutrophil elastase inhibitors sivelestat or SERPINB1 as follows: sham, OALT treated with vehicle (OALT+vehicle), OALT treated with sivelestat (OALT+sit), and OALT treated with recombinant SERPINB1 protein (OALT+rSB1). Recombinant SERPINB1 protein (100 *μ*g/kg, Sino Biological Inc., China) was administered intratracheally 24 h before operation. Sivelestat sodium hydrate (4 mg/kg, MedChemExpress, NJ, USA) was administered intravenously 1 h before operation. Samples were collected at 8 h after liver reperfusion.

All the rats received incisional infiltration of local anesthesia with 0.5% ropivacaine (1.0 mL/kg, AstraZeneca, USA) before laparotomy plus a single subcutaneous administration of ketoprofen (40 mg/kg, Sigma-Aldrich Corp., St. Louis, MO, USA) after abdominal closure for postoperative analgesia.

### 2.3. Hematoxylin-Eosin Staining

Rats were sacrificed and lung tissues were harvested at different timepoints as indicated in the experimental design. The right main bronchus was ligated, and the left lung was lavaged with physiological saline three times. The bronchial alveolar lavage fluid was collected for inflammatory cell counting. The right upper lobe was used for the determination of the dry-wet mass ratio, and the right middle and lower lobes were fixed with formaldehyde and embedded in paraffin for subsequent hematoxylin-eosin staining. Pathological changes of lung tissue were observed under a light microscope. Lung injury was scored by two pathologists according to the evaluation standard described in our previous study [20].

### 2.4. Naphthol AS-D Chloroacetate Esterase Technique

A naphthol AS-D chloroacetate esterase technique was used to label neutrophils in the lung using a specific commercial esterase kit (Sigma-Aldrich Corp., St. Louis, MO, USA) according to the manufacturer's instructions.

### 2.5. Wright-Giemsa Staining

The bronchial alveolar lavage fluid was collected for inflammatory cell counting using Wright-Giemsa's staining using a commercial kit (Sigma-Aldrich Corp., St. Louis, MO, USA) according to the manufacturer's instructions.

### 2.6. ELISA

The concentration of TNF-*α* (Nanjing Jiancheng Bioengineering Institute, Nanjing, China) in serum and lung tissue homogenates, the activities of 15-F_2t_-Isoprostane (Cayman Chemical Company) and superoxide dismutase (SOD) (Cayman Chemical Company) in lung tissue, and the concentration of NE (Cloud-Clone Crop, UA) in alveolar lavage fluid were detected by ELISA according to the manufacturer's instructions.

### 2.7. Immunofluorescence

Paraffin-embedded lung blocks were sliced into 5 *μ*m sections. ZO-1 (1 : 500, Santa Cruz Biotechnology, USA), occludin (1 : 500, Santa Cruz Biotechnology, USA), and HO-1 (1 : 1000, Novus Biologicals, USA) staining was carried out to detect protein expression in the lung by using immunofluorescence methods described in our previous study [21].

### 2.8. Statistical Analysis

At least three replicates were performed for each biological experiment. The results were expressed as mean ± SEM. The differences between the groups were analyzed by one-way ANOVA. Tukey's test was used for further comparison. Statistical analysis of all experimental data was performed using GraphPad Prism 6 software, and the results were analyzed with *p* < 0.05 as the threshold for a statistically significant difference.

## 3. Results

### 3.1. Neutrophil Infiltration in Lung Tissue after Orthotopic Autologous Liver Transplantation in Rats

**Figure 1:**
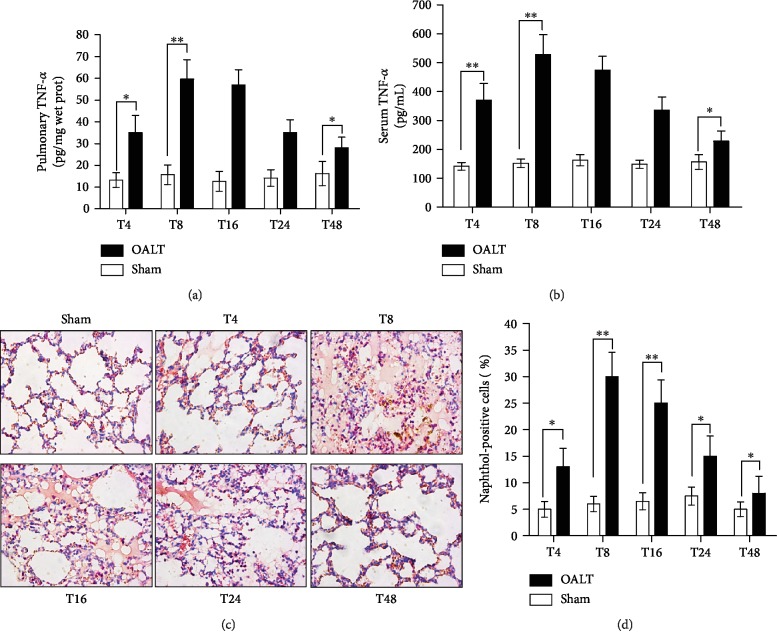
Neutrophil infiltration at different time points post-OALT. Pulmonary TNF-*α* (a) and serum TNF-*α* (b) were detected by ELISA methods. Neutrophil infiltration evidenced by naphthol staining ((c) and (d): 200x) at 4 h (T4), 8 h (T8), 16 h (T16), 24 h (T24), and 48 h (T48) after liver reperfusion onset. Each bar represents the mean ± SEM (*n* = 6 per group). ^∗^*p* < 0.05 and ^∗∗^*p* < 0.01; one-way ANOVA with Tukey's test. OALT: orthotopic autologous liver transplantation.

Neutrophil infiltration plays an important role in ALI induced by LT [17]. As shown in Figures 1(a) and 1(b), pulmonary and serum TNF-*α* were significantly increased from 4 h after OALT and peaked at 8 h after OALT. Moreover, the infiltration of neutrophils in the injured lung tissue was further examined along with the marker related to neutrophil infiltration. The results of naphthol esterase staining showed that the infiltration of lung tissue by neutrophils was obvious after LT, and the number of naphthol esterase-positive cells (Figures 1(c) and 1(d)) peaked at 8 h (T8) after OALT and gradually decreased after 24 h (T24) (*p* < 0.05 vs. sham). All these indicate that severe inflammatory infiltration in the lung increased and peaked 8 h after OALT. Thus, the timepoint of 8 h after OALT was chosen in the subsequent experiments.

### 3.2. Neutrophil Elastase Inhibitors Reduce Lung Tissue Neutrophil Infiltration after Liver Transplantation

**Figure 2:**
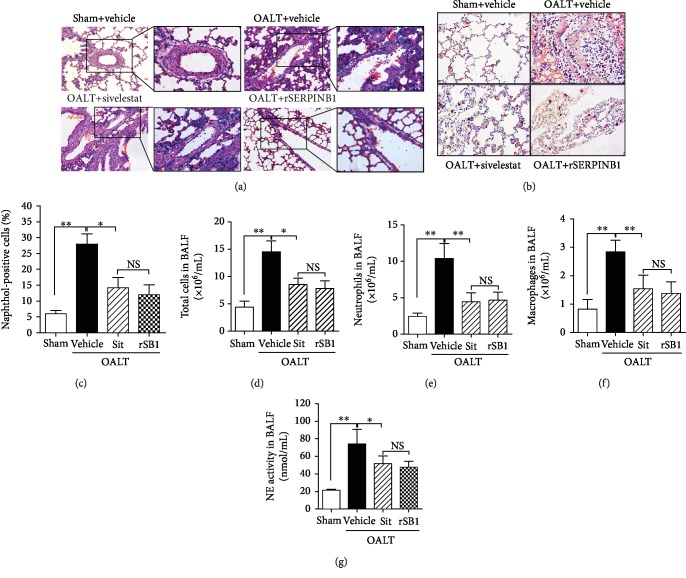
Neutrophil infiltration attenuated by the neutrophil elastase inhibitor in injured lung post-OALT. Neutrophil infiltration was detected at 8 h post-OALT in the lung of the rat, which was given rSERPINB1 24 h or sivelestat sodium 1 h before surgery. Neutrophil infiltration was clearly observed via lung H&E ((a): 100x and 400x) staining and naphthol staining ((b) and (c): 200x). Moreover, the numbers of total cells (d), neutrophils (e), and macrophages (f) were counted in the collected BALF using Wright's staining. NE activity (g) in BALF was determined via ELISA. Each bar represents the mean ± SEM (*n* = 6 per group). ^∗^*p* < 0.05 and ^∗∗^*p* < 0.01; one-way ANOVA with Tukey's test. rSERPINB1 (rSB1): recombinant SERPINB1 protein; Sit: sivelestat; BALF: bronchoalveolar lavage fluid; OALT: orthotopic autologous liver transplantation.

We further studied the effect of NE inhibitors on neutrophil infiltration. Hematoxylin-eosin staining showed that high infiltration by inflammatory cells in lung tissue was observed at 8 h after OALT in rats (Figure 2(a)). The total number of cells and the number of neutrophils in the alveolar lavage fluid were detected by Wright's staining, which showed that the number of neutrophils significantly increased (*p* < 0.01 vs. sham) (Figures 2(d)–2(f)). Moreover, naphthol esterase staining (Figures 2(b) and 2(c)) showed high neutrophil infiltration in lung tissues at 8 h (*p* < 0.05 vs. sham).

Furthermore, by intravenous administration of sivelestat sodium hydrate in the OALT+sit group or intratracheal administration of recombinant human SERPINB1 protein in the OALT+rSB1 group, we found that both NE inhibitors significantly attenuated neutrophil infiltration and NE (Figure 2(g)) in lung tissues. These were accompanied by decreases in the total number of cells in alveolar lavage, the number of neutrophils, and the number of macrophages, as well as the number of naphthol esterase-positive cells in the lung tissue (*p* < 0.05 vs. OALT+vehicle).

### 3.3. Neutrophil Elastase Inhibitors Attenuate Oxidative Stress Damage and Restore Alveolar Barrier Function

**Figure 3:**
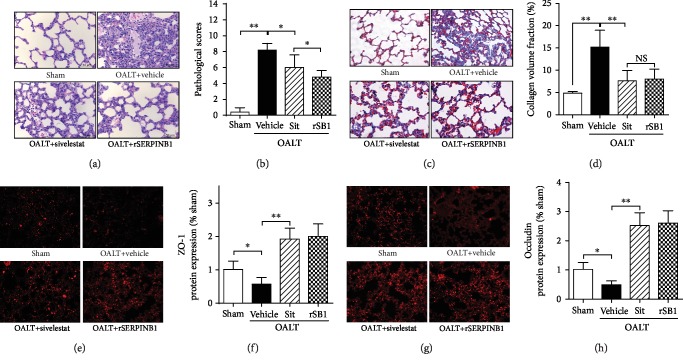
Attenuation of ALI, reduction of pulmonary fibrosis protein generation, and restoration of alveolar barrier function by neutrophil elastase inhibitor. Lung injury and lung tissue fibrosis-associated protein were evaluated using H&E staining ((a) and (b): 400x) and Masson's staining ((c) and (d): 200x) at 8 h post-OALT in the rat lung which was given rSERPINB1 24 h or sivelestat sodium 1 h before surgery. Lung ZO-1 ((a) and (b): 200x) and occludin ((c) and (d): 200x) protein expressions were assayed by immunofluorescence at 8 h post-OALT in the rat lung which was given rSERPINB1 24 h or sivelestat sodium 1 h before surgery. Each bar represents the mean ± SEM (*n* = 6 per group). ^∗^*p* < 0.05 and ^∗∗^*p* < 0.01; one-way ANOVA with Tukey test. rSERPINB1 (rSB1): SERPINB1 recombinant protein; Sit: sivelestat; OALT: orthotopic autologous liver transplantation.

We further evaluated the effects of SERPINB1 recombinant protein and sivelestat sodium hydrate on pathological damage and alveolar barrier function in lung tissue. The results of hematoxylin-eosin staining (Figures 3(a) and 3(b)) showed that SERPINB1 recombinant protein and sivelestat sodium hydrate alleviated the pathological damage of lung tissues to different extents. Compared with the control group, the SERPINB1 recombinant protein group and the sivelestat sodium hydrate group showed lesser infiltration of lung tissue inflammation, lower alveolar intraluminal exudation and alveolar stenosis, improved pulmonary interstitial hemorrhage, thinned alveolar septum, and lower proportion of lung parenchyma.

Pathological scoring results of lung tissue damage showed that the SERPINB1 recombinant protein group had a lower injury score than the sivelestat sodium hydrate group (*p* < 0.05 vs. OALT+sivelestat). The results of Masson's staining of lung tissue showed that both SERPINB1 recombinant protein and sivelestat sodium hydrate were effective in reducing collagen fiber content (Figures 3(c) and 3(d)).

Similarly, tissue immunofluorescence staining showed the expression of tight junction proteins ZO-1 (Figures 3(e) and 3(f)) and occludin (Figures 3(g) and 3(h)), reflecting that the alveolar barrier function was significantly higher in the SERPINB1 recombinant protein and the sivelestat sodium hydrate group than in the control group; however, there was no statistical difference between the two groups.

### 3.4. Neutrophil Elastase Inhibitors Reduce Lung Tissue Oxidation after Liver Transplantation

**Figure 4:**
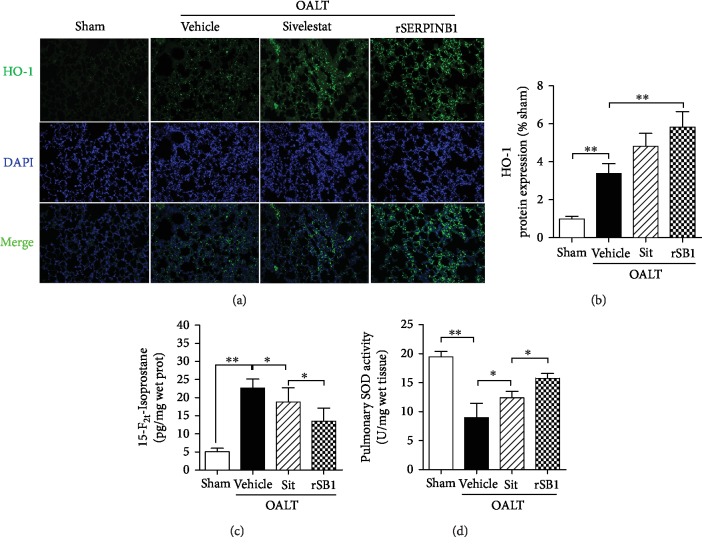
Effects of neutrophil elastase inhibitor on pulmonary oxidative stress post-OALT. Lung oxidative stress was evaluated at 8 h post-OALT in the lung of the rat which was given rSERPINB1 for 24 h or sivelestat sodium hydrate for 1 h before surgery. HO-1 expression in the lung was detected by immunofluorescence ((a) and (b): 200x). Lung 15-F_2t_-isoprostane (c) and SOD (d) activities were determined via ELISA. Each bar represents the mean ± SEM (*n* = 6 per group). ^∗^*p* < 0.05 and ^∗∗^*p* < 0.01; one-way ANOVA with Tukey test. rSERPINB1 (rSB1): SERPINB1 recombinant protein; Sit: sivelestat; OALT: orthotopic autologous liver transplantation.

To explore the mechanism of NE inhibitors on protecting the lung against ALI, we examined the oxidative stress-related proteins in lung tissues. The immunofluorescence staining of antioxidant enzyme HO-1 (Figures 4(a) and 4(b)) in lung tissue showed that both SERPINB1 recombinant protein and sivelestat sodium hydrate could significantly increase the expression of HO-1 in lung tissue. We also detected the markers of oxidative stress and found that the level of 15-F_2t_-isoprostane (Figure 4(c)) was increased in rat lungs following OALT, accompanied with a decrease in SOD activities (Figure 4(d)). After treatment with SERPINB1 recombinant protein or sivelestat sodium hydrate, the level of 15-F_2t_-isoprostane was decreased and the SOD activities were elevated.

## 4. Discussion

Our findings in the present study provide new insights into the prospective effects of NE inhibitors on ALI. NE, a serine proteinase released from neutrophils, participates in the pathogenic process of tissue injury [22, 23]. In the current study, we showed that inflammation and NE infiltration were increased in the lung tissues after OALT that were associated with increased oxidative stress and severe lung injury. Further, we demonstrated that NE inhibitors, sivelestat sodium hydrate and human SERPINB1 recombinant protein, protected against OALT-induced ALI by improving pulmonary antioxidant properties and reducing damage to alveolar epithelial cells. These findings provide further evidence that NE inhibitors could serve as effective therapeutic agents for LT-related ALI.

Inflammation and neutrophil infiltration lead to severe lung injury after LT. Neutrophil infiltration occurred in graft liver early after transplantation, which may be initiated by the amount of neutrophil chemokines, including tumor necrosis factor-*α* (TNF-*α*), interleukin-1*α* (IL-1*α*) and IL-1*β*, activated complement factors, platelet-activating factor and CXC chemokines, macrophage inflammatory protein-2 (MIP-2), and cytokine-induced neutrophil chemoattractant (CINC-1) [24–26]. In the current study, we found that serum TNF-*α* levels were significantly elevated during early stages of LT, indicating that TNF-*α* might act as the main chemokine in recruiting circulating neutrophils to the liver. Next, neutrophils migrate from the liver to the lung via the postcava, with most being detained in the lung and subsequently being phagocytized by alveolar macrophages to limit tissue inflammatory injury [27]. We also found that neutrophils infiltrated in the lung may lead to more severe lung injury following OALT, which indicates that the migrating neutrophils may release some granules to avoid macrophage clearance or may directly attack the alveolar epithelial cells.

NE released from neutrophils caused further lung oxidative stress after LT. In our study, we found that BALF NE significantly increased following OALT, which was closely related to the filtration of neutrophils, suggesting that NE may act as a mediator during neutrophil infiltration-related lung injury during LT. Similarly, a recent study also found that NE promoted ALI induced by lipopolysaccharide stimulation, which may be related to a mechanism of matrix metalloproteinase-9 reduction [28]. Furthermore, our results showed that neutrophil infiltration and NE release contributed to pulmonary barrier dysfunction via aggravating lung oxidative stress, similar to the findings of Aoshiba et al., which showed that the treatment of bronchial epithelial or lung fibroblast cells with the serine protease NE increased mitochondrial and cytoplasm ROS levels [29].

Inhibition of NE activity contributed to lung protection after LT. Sivelestat sodium hydrate, a synthetic, potent, and selective inhibitor of human NE, has been identified as a therapeutic drug against ALI [30, 31]. In the current study, we compare sivelestat sodium hydrate with another NE inhibitor, recombinant human SERPINB1 protein, in the treatment of OALT-induced ALI. In our previous study, we identified SERPINB1 as a therapeutic agent via proteomic technology using the animal lung sample following OALT [16]. Although endogenous SERPINB1 was revealed to be increased during OALT-induced ALI, lung injury still existed and developed gradually, indicating that the amount of endogenous SERPINB1 was insufficient against injury-related NE release. Recombinant SERPINB1 protein was given before surgery to elevate the concentration of SERPINB1 in the lung and subsequently inhibited NE combined with the original SERPINB1. SERPINB1 directly links to the serine residue of NE and inhibits its function, while sivelestat sodium hydrate inhibits NE by reducing the production and release of NE from neutrophils [32]. In the present study, we found that both sivelestat sodium hydrate and recombinant human SERPINB1 protein could effectively improve pulmonary function following OALT, indicating that inhibiting NE either indirectly or directly provides a lung-protective effect following LT.

Of note, there still remain limitations to the current study. As neutrophil apoptosis plays a key role in inflammation resolution [33] and the NE inhibitor SERPINB1 has been identified as an antiapoptosis protein [34], possible mechanisms by which the NE inhibitors affect neutrophil apoptosis during ALI induced by OALT need to be investigated in the future. Moreover, the current study showed that both NE inhibitors, sivelestat sodium hydrate and SERPINB1, have antioxidant properties; however, the difference between the two NE inhibitors has not been clarified and needs further study.

**Figure 5:**
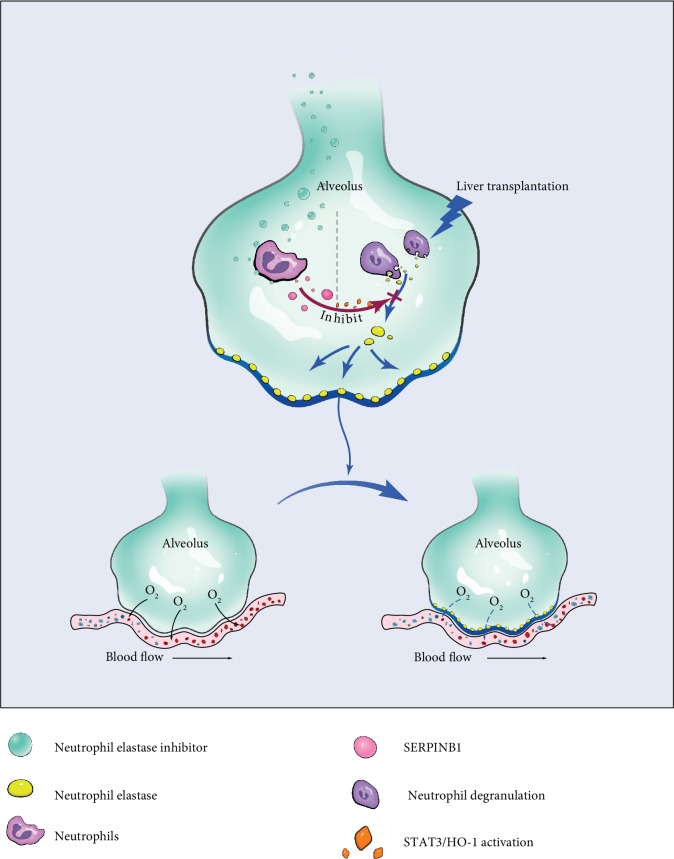
Schematic representation of the effect of neutrophil elastase inhibitors suppressing oxidative stress in alveolar epithelial cells during liver transplantation. Liver transplantation injury induces neutrophil infiltration in alveoli and neutrophil degranulation and neutrophil elastase release which lead to pulmonary edema and dysfunction. Exogenous (sivelestat) and endogenous (SERPINB1) neutrophil elastase inhibitors activate antioxidant enzymes and attenuate lung damage by way of antioxidative stress.

In summary, the NE inhibitors sivelestat sodium hydrate and SERPINB1 both suppressed NE, attenuated lung neutrophil infiltration, and finally, restored pulmonary barrier function, which is related to the reduction of pulmonary oxidative stress (Figure 5).

## Data Availability

The datasets generated and/or analyzed during the current study are available from the corresponding authors on reasonable request.
